# Chelant Enhanced Solution Processing for Wafer Scale Synthesis of Transition Metal Dichalcogenide Thin Films

**DOI:** 10.1038/s41598-017-06699-7

**Published:** 2017-07-25

**Authors:** Robert Ionescu, Brennan Campbell, Ryan Wu, Ece Aytan, Andrew Patalano, Isaac Ruiz, Stephen W. Howell, Anthony E. McDonald, Thomas E. Beechem, K. Andre Mkhoyan, Mihrimah Ozkan, Cengiz S. Ozkan

**Affiliations:** 1 Materials Science and Engineering Program, Department of Mechanical Engineering, University of California Riverside, Riverside, CA 92521 USA; 2 Department of Chemistry, Department of Electrical Engineering, University of California Riverside, Riverside, CA 92521 USA; 3 Department of Chemical Engineering & Materials Science, University of Minnesota, Minneapolis, MN 55455 USA; 4 Sandia National Laboratories, Albuquerque, NM 87123 USA

## Abstract

It is of paramount importance to improve the control over large area growth of high quality molybdenum disulfide (MoS_2_) and other types of 2D dichalcogenides. Such atomically thin materials have great potential for use in electronics, and are thought to make possible the first real applications of spintronics. Here in, a facile and reproducible method of producing wafer scale atomically thin MoS_2_ layers has been developed using the incorporation of a chelating agent in a common organic solvent, dimethyl sulfoxide (DMSO). Previously, solution processing of a MoS_2_ precursor, ammonium tetrathiomolybdate ((NH_4_)_2_MoS_4_), and subsequent thermolysis was used to produce large area MoS_2_ layers. Our work here shows that the use of ethylenediaminetetraacetic acid (EDTA) in DMSO exerts superior control over wafer coverage and film thickness, and the results demonstrate that the chelating action and dispersing effect of EDTA is critical in growing uniform films. Raman spectroscopy, photoluminescence (PL), x-ray photoelectron spectroscopy (XPS), Fourier transform infrared spectroscopy (FTIR), atomic force microscopy (AFM) and high-resolution scanning transmission electron microscopy (HR-STEM) indicate the formation of homogenous few layer MoS_2_ films at the wafer scale, resulting from the novel chelant-in-solution method.

## Introduction

2D materials have been intensively studied for the past few decades with ever-growing interest. Large area growth of 2D materials remains a challenge that must be overcome for commercial applications^1^. 2D layered TMDs such as molybdenum disulfide (MoS_2_) and tungsten disulfide (WS_2_) have received much attention due to their electronic and optical properties^2–4^. Graphene has played a central role in 2D materials research due to its high carrier mobility but its lack of a band gap hinders its potential for applications in the IC industry^5–12^. Unlike graphene, MoS_2_ possesses a film thickness dependent bandgap in the optical range (~1.3 eV bulk, ~1.8 eV monolayer)^13, 14^. The change in bandgap energy is accompanied by a transition from indirect in bulk to direct in single layer MoS_2_, which has been observed using photoluminescence (PL) spectroscopy^15^. These properties make thin-layer MoS_2_ films suitable for field effect devices and optical applications, which further accentuate the need to achieve thin layered films over large areas. Here, we report an advancement towards full wafer scale synthesis of MoS_2_ by using a facile solution based synthesis.

Solution based methods have been emerging as novel techniques for obtaining wafer scale 2D thin films. Many have taken advantage of either dip coating or spin coating but so far uniformity across large areas have been difficult to achieve. Putz *et al*. first reported such a process using ammonium thiomolybdate ((NH_4_)_2_MoS_4_), which could be utilized for thin film applications as a single precursor with no need for sulfur addition^16–18^. Liu *et al*. produced MoS_2_ films by dip-coating silicon dioxide (SiO_2_) substrates in a solution prepared by dissolving (NH_4_)_2_MoS_4_ in dimethylformamide (DMF)^19^. This method, however, is difficult to scale up due to the unpredictability of dip coating and precise control of layer thickness. An alternative method -spin coating- has been shown as a more efficient way to obtain controlled layers based on the solution concentration viscosity and spin coating parameters^20^. George *et al*. performed a spin coating process by dissolving (NH_4_)_2_MoS_4_ in n-methylpyrollidone (NMP) and obtained various thicknesses based on solution processing^20^, which provided a new approach to obtain wafer scale TMD layers. However, further refinement to reduce the density of surface defects, dewetted areas, and to improve large area uniformity was still needed^20^.

Oxide precursors such as molybdenum trioxide (MoO_3_) and tungsten trioxide (WO_3_) have alternatively been utilized to obtain MoS_2_, WS_2_ and other TMD domains and layers^6, 21–23^. In these techniques, mostly triangular domains associated with CVD processing have been observed but their dimensions were very much limited^6^. Similar research done towards large area growth or large grain growth using either oxide powder or hydrothermally synthesized oxides indicated slightly larger features, but not exceeding 200 μm across the domains with most reports averaging in the range 10–20 μm^5, 6, 24^. Alternatively, work done by Kang *et al*. has been promising for large area synthesis of 2D materials utilizing MOCVD processing which could be scaled up and implemented at an industrial scale, but yet to be realized for wafer scale electronics applications^25^. Research and development for growing 2D materials will still be ongoing in the coming years to develop further novel approaches.

**Figure 1:**
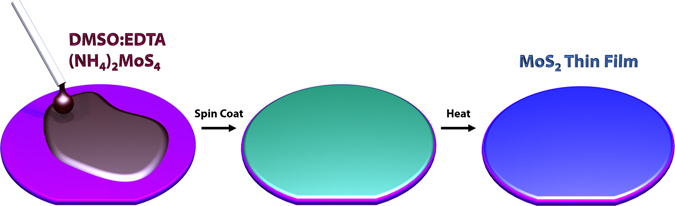
Schematic of spin coating process. Solution application onto the wafer is illustrated along with color observation of after spin coating and heating process.

Here, we demonstrate a uniform non-sulfur assisted method of wafer-scale synthesis of MoS_2_ films using a chelating agent. Figure 1 shows a simple schematic of the chelant-enhanced solution based synthesis of MoS_2_ thin films. A solution of ethylenediaminetetraacetic acid (EDTA) in Dimethylsulfoxide (DMSO) was first prepared before dissolving the (NH4)2MoS4 precursor. To enhance the wettability of our substrates, an RCA clean was performed followed by an HF wash. Large area films were synthesized by tuning and optimizing the ratio of precursor concentrations and spin coating parameters. Homogeneity of as synthesized MoS_2_ films were characterized by Raman spectroscopy, Photoluminescence (PL), X-ray photoelectron spectroscopy (XPS), Fourier transform infrared spectroscopy (FTIR) atomic force microscopy (AFM) and high-resolution scanning transmission electron microscopy (HR-STEM).

## Results

**Figure 2:**
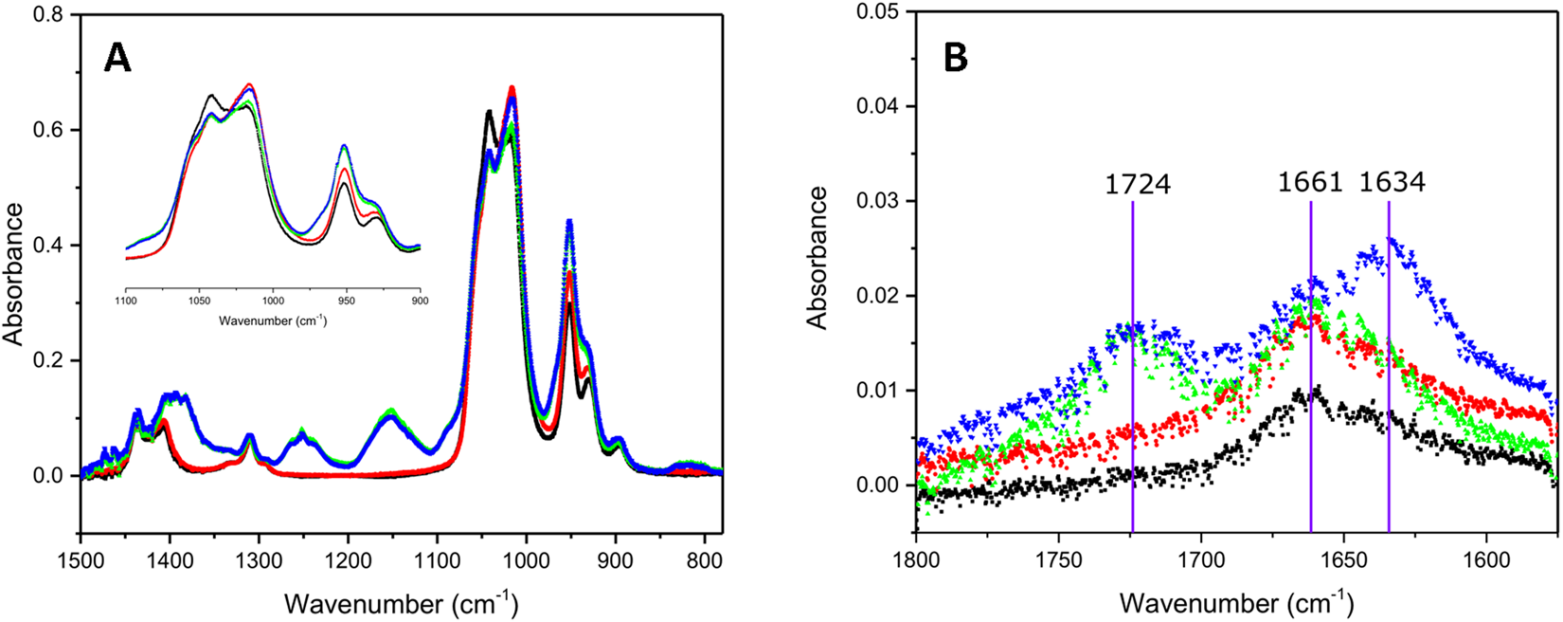
FTIR-ATR data of (NH_4_)_2_MoS_4_ solutions as seen in **A** and **B**. Black represents the solvent DMSO, Red represents 240 mg of (NH_4_)_2_MoS_4_ in 5 mL DMSO, Green represents 0.146 EDTA in 5 mL DMSO, Blue squares represents 0.146 g EDTA 240 mg of (NH_4_)_2_MoS_4_ in 5 mL DMSO.

FTIR data from the prepared solutions were obtained to study potential molecular interactions. In Fig. 2, the black curves represent pure DMSO solution while red curves represent (NH_4_)_2_MoS_4_ dissolved in DMSO; curves in green represent EDTA dissolved in DMSO; and curves in blue represent EDTA and (NH_4_)_2_MoS_4_ dissolved in DMSO. Figure 2A and B shows the IR ν_SO_ spectral region for EDTA, molybdenum precursor salt and a combination of these species in DMSO solutions. Altogether, the data identifies the regions exhibiting significant observable changes. The measurements are primarily focused on the DMSO’s S=O stretching regions and their changes in the presence of EDTA salt and a combination of external intermolecular interaction stimuli. The primary focus was the S=O region spectra since C-H region were relatively stable in spectral shape and position for DMSO even when the molybdenum salt was introduced. Figure 2A indicates noticeable changes in the spectral pattern at 1041 cm^−1^ and 1018 cm^−1^ in the presence of any other additives in DMSO. The intensity of the band at 1041 cm^−1^ reduces while the intensity of the band at 1018 cm^−1^ increases. The additives do not change the frequency for 1040 cm^−1^ and shows a slight red shift from 1018 cm^−1^ to 1016 cm^−1^. No noticeable intensity changes are observed for the CH_3_ peak at 950 cm^−1^ in the presence of EDTA or only molybdenum precursor salt addition. At 697 cm^−1^ and 667 cm^−1^, no noticeable changes were observed for both intensity and frequency of the vibrational bands.

The IR data provided offers an explanation for the interactions taking place in the pre-spun mixtures. Intensity changes in the region 1041 cm^−1^ to 1018 cm^−1^ indicate interactions of additives with the primary solvent DMSO, including changes in hydrogen bonding interactions with the solvent^26^. The high absorbance in this range suggests that DMSO-DMSO interactions are the strongest interactions in this system^26^. A more subtle effect is observed due to EDTA in this mixture, which shows a stretch behavior characteristic of aliphatic amines and carboxylic acid groups interacting with dissolved metal cations in the mixture^27, 28^.

The IR spectra of ketones and carboxylic acids are generally characterized by absorptions in the ranges 1750–1660 cm^−1^ and 1300–1200 cm^−1^ representing the C=O and C–O bonds, respectively^28^. The carboxylate ions have antisymmetric modes in the range 1650–1510 cm^−1^ and relatively strong symmetric COO^−^ stretching in the 1400–1280 cm^−1^ range. Stretching vibrations at 1661 cm^−1^ in the DMSO-EDTA solution are assigned to the EDTA`s asymmetric COO^−^ stretching and the high intensity peak at 1400 cm^−1^ is attributed to the symmetrical COO^−^ stretching. The peaks in the 1360–1100 cm^−1^ region are attributed to NH^+^ stretching. The addition of a molybdenum precursor salt shifts the asymmetric COO^−^ stretch from 1661 cm^−1^ to 1634 cm^−1^ ^27, 28^. Coordination modes of these carboxylic acids are generally in three common groups and can be distinguished in the IR spectra by their separations between the carboxylate stretch bands (Δν). The band separations are in the range of 350–500 cm^−1^ for monodentate binding, 150–180 cm^−1^ for bridging, and 60–100 cm^−1^ for chelating^28^. A separation, Δν, was observed to be 228 cm^−1^ in this experimental setup.

The result of ATR-FTIR studies overall indicate that the addition of a molybdenum salt or EDTA separately results in the breaking of S=O. Hydrogen bonding thus disturbs the dimer-like or larger polymer-like hydrogen bonding that drives the self-assembly. Addition of all these species at once showed similar a trend for the S=O stretching region, however, a combination of all indicated significant changes for the COO^−^ asymmetric stretching region. Overall, our observations suggest that EDTA and molybdenum salt solutions in DMSO form a network, which is primarily driven by hydrogen bonding in which EDTA forms a mixture of monodentate, and bridging interactions with the molybdenum salt.

**Figure 3:**
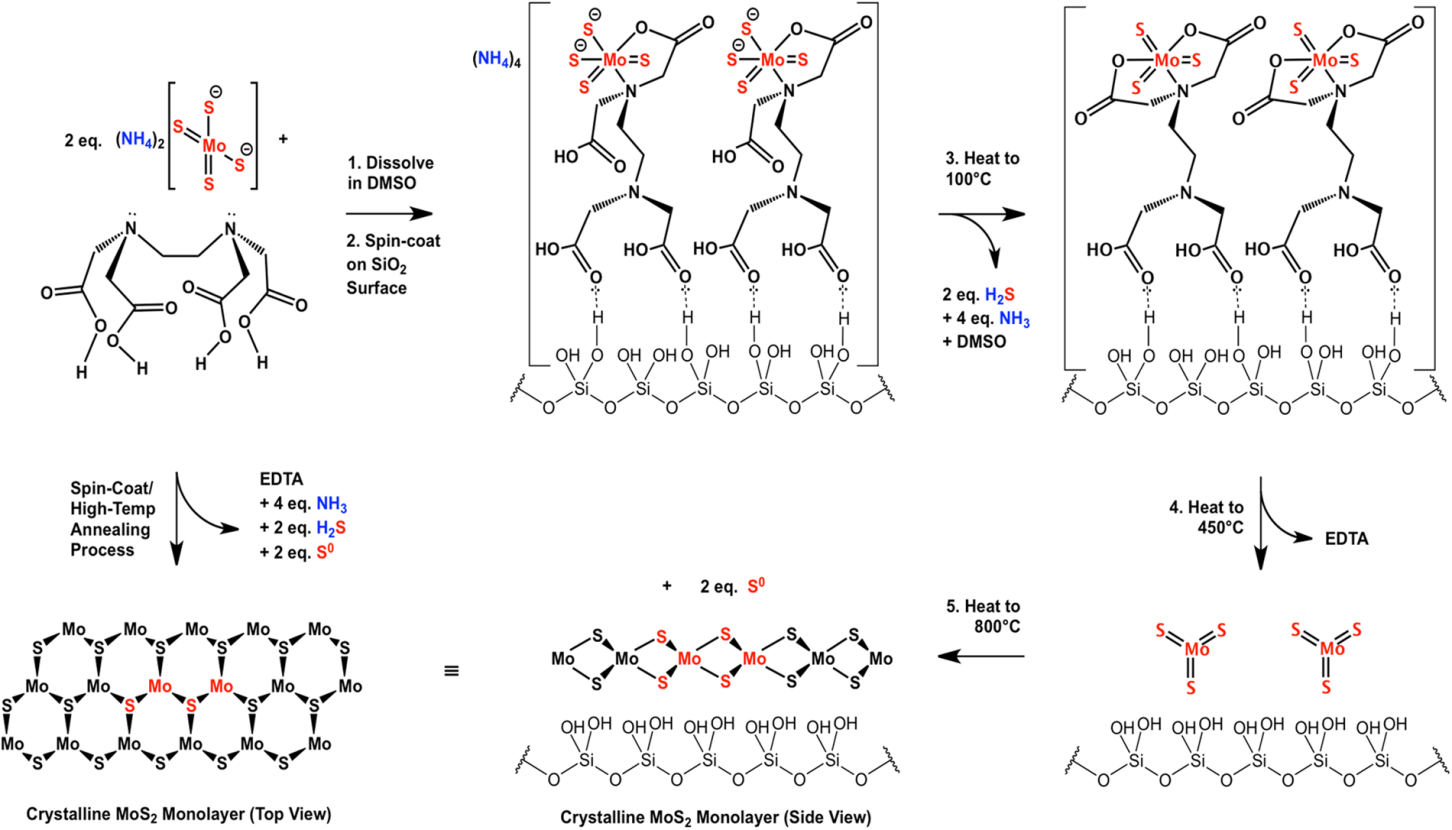
Mechanism for the synthesis of crystalline MoS_2_.

The demonstrated advantages of EDTA in this synthetic technique when added the metal sulfide precursor in DMSO come from superior wettability on the hydroxylated surface which is observed in the schematic provided in Fig. 3. Tetrathiomolybdate coordinates with EDTA as soon as the solution is prepared, which is indicated by an upfield shift of the EDTA C=O stretching from 1724 cm^−1^ to 1634 cm^−1^ ^28^. The lowered frequency of the upfield C=O stretch can be explained by a σ-donation of the acid group of EDTA to the cationic Mo(VI) metal center^28^. The IR spectrum of MoS_4_^2−^ in DMSO with EDTA is also indicative for the presence of uncoordinated carboxylic acid groups as a consistent peak at 1724 cm^−1^ (Fig. 2B) for both EDTA, and EDTA with metal sulfide and DMSO solutions. The presence of both coordinated and uncoordinated carboxylic groups could enhance the interaction with the silanol-functionalized silica surface, allowing this solution to act as an effective vehicle to evenly coat the wafer with the Mo(VI) sulfide precursor^27^.

**Figure 4:**
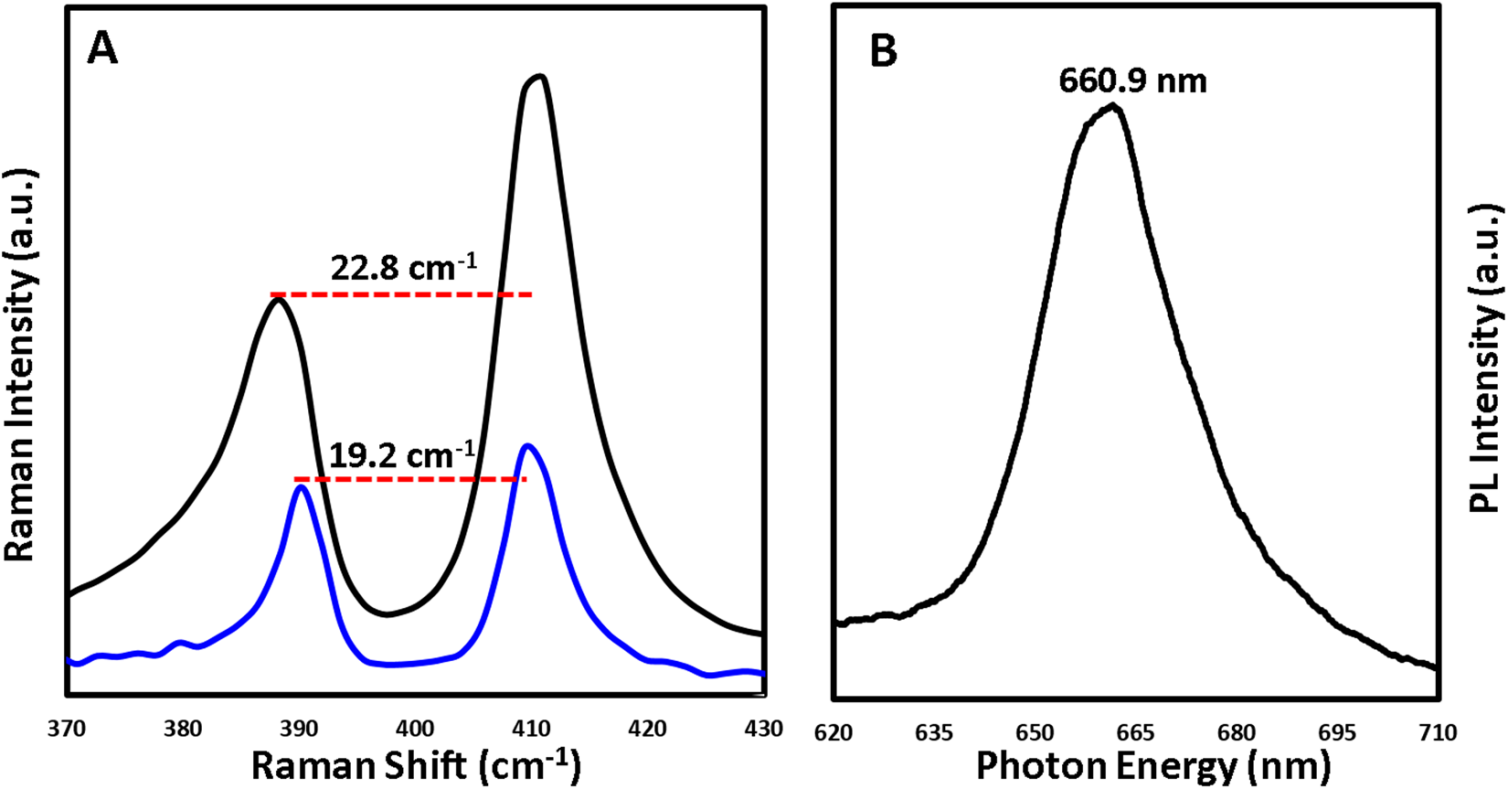
(**A**) Raman spectra of monolayer and few layers of MoS_2_. (**B**) PL characterization of MoS_2_ thin films.

Raman and PL spectroscopies allow the measurement of sample thickness and crystal quality. The frequency difference between the E^1^_2g_ and A_1g_ mode peaks, shown in Fig. 4A, indicates the presence of few-layer and monolayer MoS_2_. Raman shifts depicted by the black curve have a peak to peak distance of 22.8 cm^−1^ corresponding to few layer MoS_2_^6, 19, 20, 29, 30^. Raman shifts depicted by the color blue have a E^1^_2g_ and A_1g_ peak to peak distance of 19.2 cm^−1^ corresponding to monolayer MoS_2_^6, 19, 20, 29, 30^. This assignment is further confirmed by PL measurements taken in the same region (Fig. 4B), which exhibits a peak response near 660.9 nm (1.87 eV)^6, 19, 20, 29, 30^.

**Figure 5:**
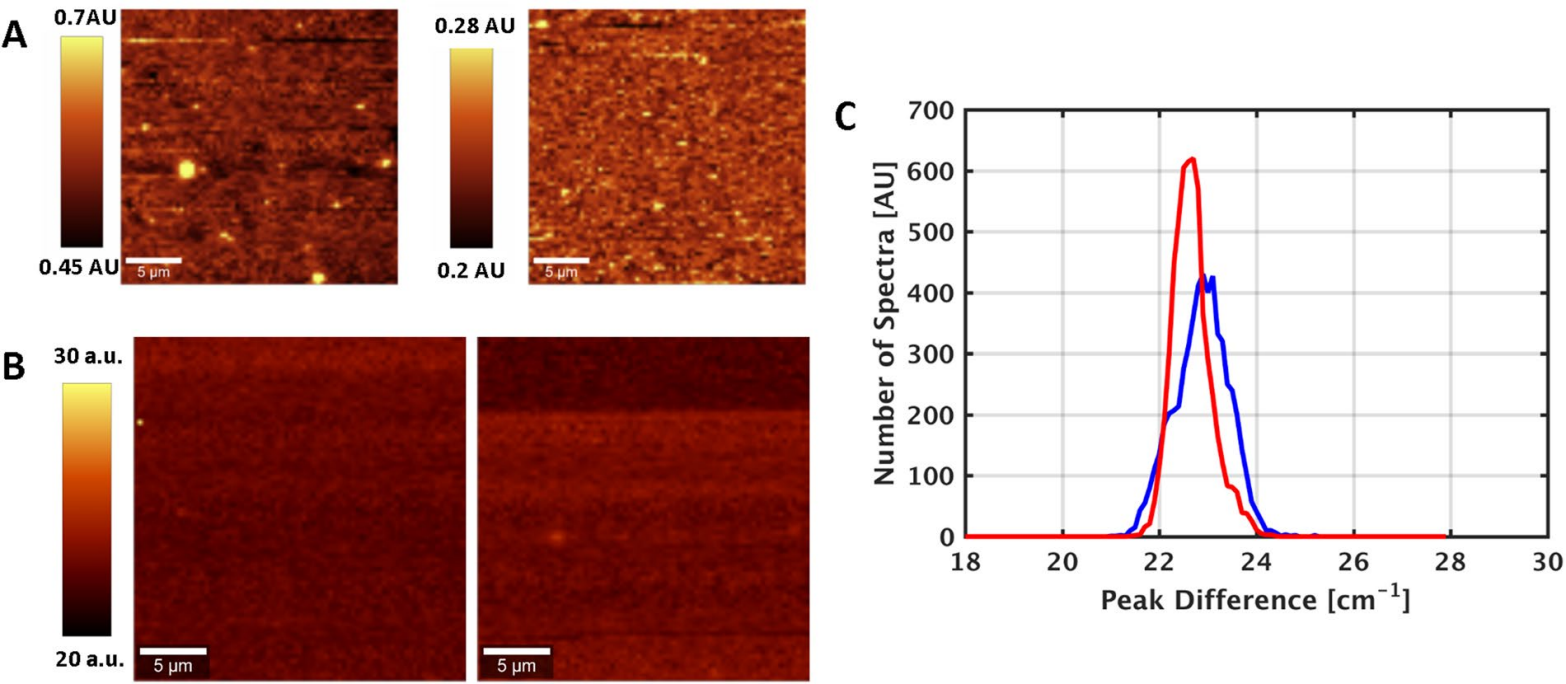
(**A**) Peak intensity ratio of the MoS_2_ (E1) peak to silicon mode. (**B** and **C**) Peak difference map between the A_1g_ and the E^1^_2g_ peak.

The uniformity and consistency of the sample was tested using Raman mapping over a 25 × 25 micron area with a spectrum acquired every 333 nm. Shown in Fig. 5A are the resulting images of two separate regions composed from the peak intensity ratio of the MoS_2_ (E^1^_2g_) peak to the triply degenerate silicon mode at 520 cm^−1^. These images confirm the presence of MoS_2_ over large areas while the small variation of intensity ratios demonstrates the relative uniformity of the film. While monolayer regions were observed intermittently primarily at the center of the wafer, the Raman images show that thickness varies across the wafer due to the nature of the spin coating process. To further probe the uniformity of the MoS_2_ films, the difference between the A_1g_ and the E^1^_2g_ peaks were mapped out for the same two regions and are displayed in Fig. 5B along with their histograms in Fig. 5C. Full-width at half-max (FWHM) of the distributions are on the order of 1 cm^−1^, which corresponds to the spectral resolution of the measurement system, indicating a relatively uniform film.

**Figure 6:**
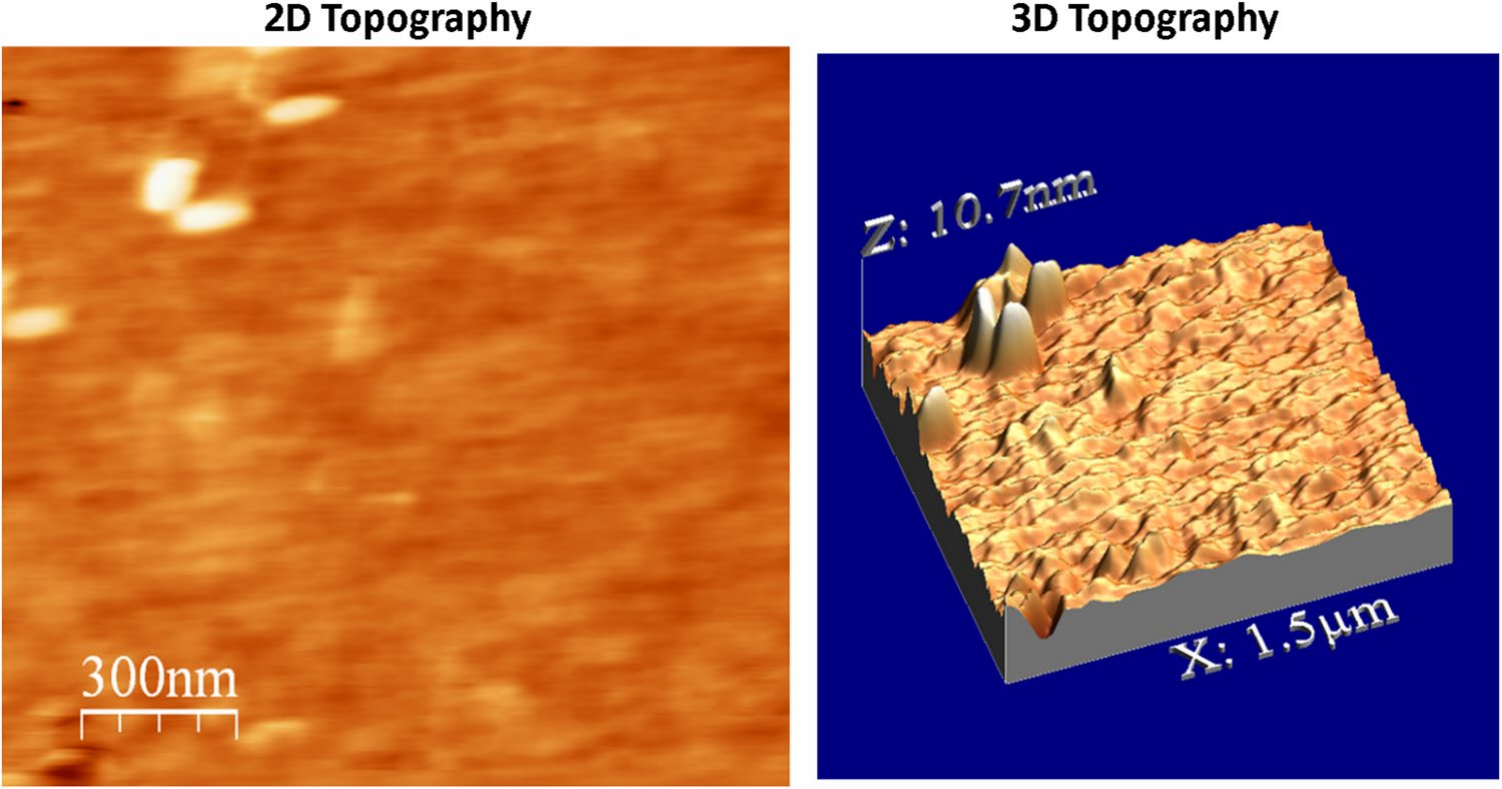
AFM surface studies of the MoS_2_ layers.

Uniformity was further examined using atomic force microscopy (AFM) across a 1.5 μm^2^ region of the film. Figure 6 shows the resulting 2D scan/mapping and a 3D profiling of the same region. The topography is relatively uniformly interspersed with a few topographic pillars, which are most likely MoS_2_ as Raman imaging shows small dots of high MoS_2_ intensity (See Fig. 5A). The overall surface roughness of the film was measured to be 0.7 nm. No obvious holes are present in the topography map or the 3D profile, indicative of complete film coverage across the substrate. There are no large step heights present in the scan beyond the few pillars providing further evidence of uniformity over the micron scale.

**Figure 7:**
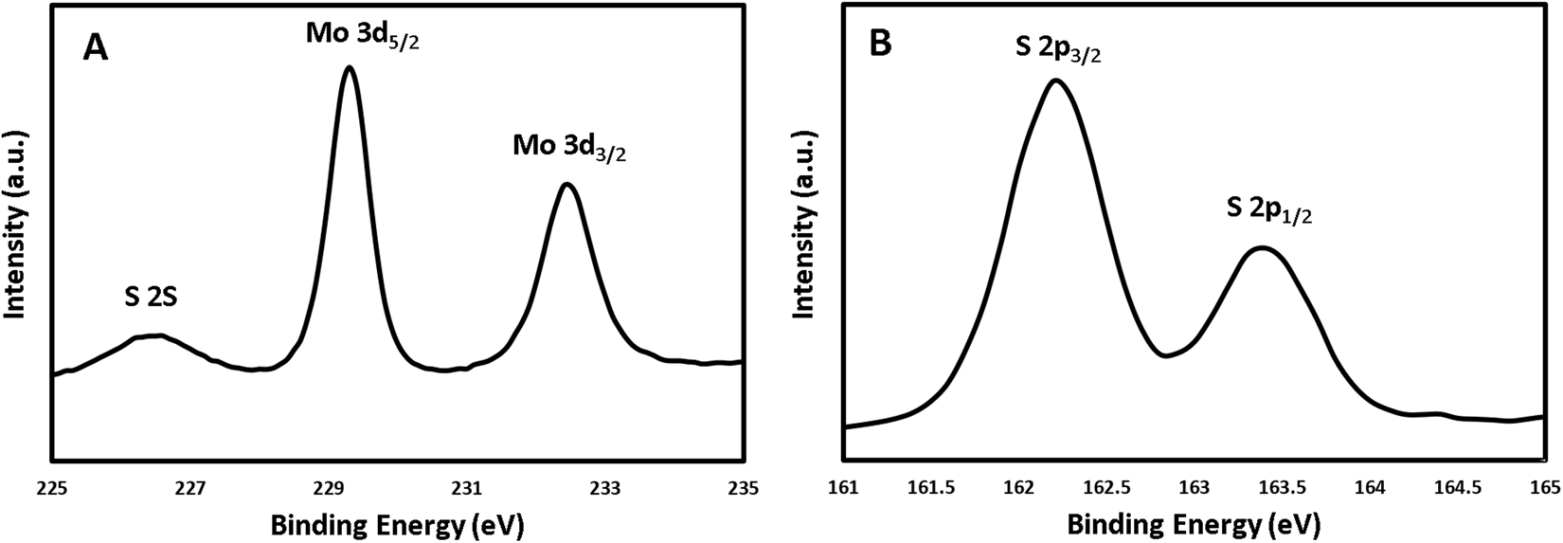
(**A**) XPS data corresponding to the Mo binding energy. (**B**) XPS data corresponding to the S binding energy.

XPS was used to determine the chemical composition of the as synthesized MoS_2_ films. Figure 7A shows the Mo binding energy exhibiting a peak for the Mo3d_5/2_ at 229.3 eV; at 232.4 eV corresponding to the Mo 3d_3/2_; and at 226.6 eV corresponding to the sulfur peak (S 2S) binding energy^19, 20, 29, 30^. These characteristics are a match to the Mo^4+^ state, noted in previous investigations^19, 20, 29, 30^. Binding energy for the spin orbit couple S 2p_3/2_ and S2p_1/2_ are 162.2 eV and 163.4 eV, respectively, as shown in Figure 7B ^19, 20, 29, 30^. The uniformity and intensity of the peaks validate the quality of the material and are consistent with the reported values for MoS_2_ crystal structure.

**Figure 8:**
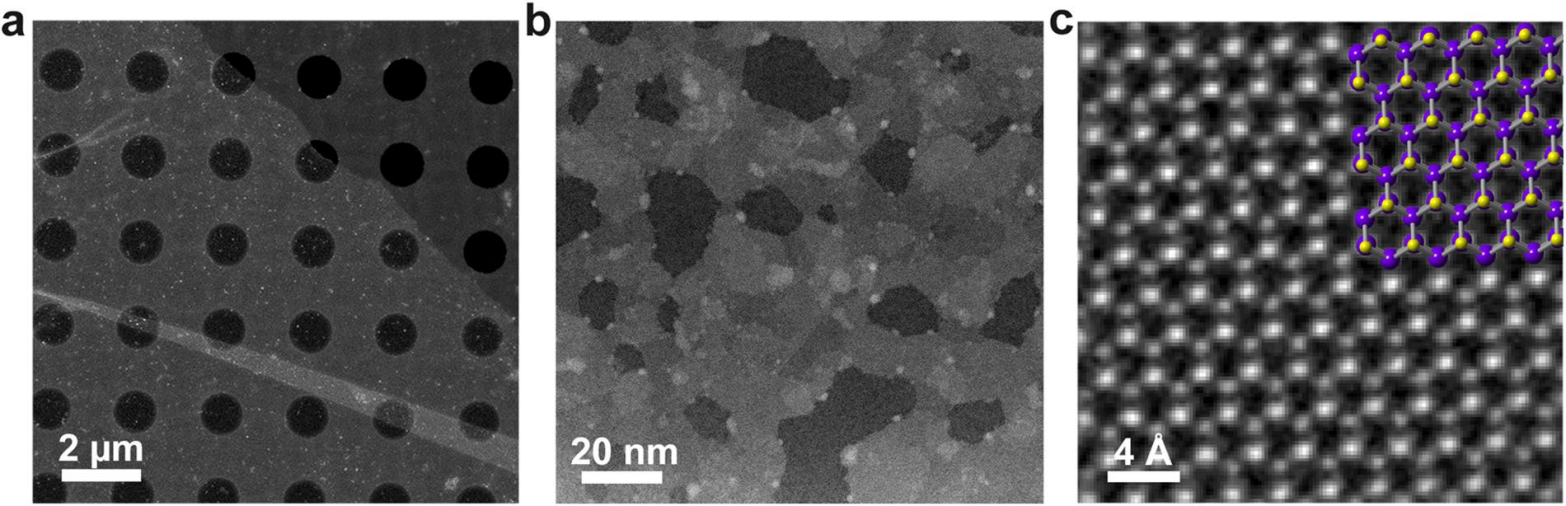
ADF-STEM images of synthesized MoS_2_ at various magnifications (**a**) Low magnification image showing a large synthesized and transferred continuous MoS_2_ film. (**b**) Intermediate magnification image showing regions of various thicknesses as indicated by differences in intensity. (**c**) High magnification image showing the hexagonal atomic structure of MoS_2_.

To characterize the atomic structure of the solution grown film, a representative sample was transferred onto a quantifoil TEM grid (please see experimental details) in preparation for STEM imaging. Figure 8a shows a low magnification annular dark field (ADF)-STEM image of a transferred film (brighter regions) on top of the quantifoil grid (darker regions) where the patterned black circular spots are holes in the grid. At this magnification, the large continuous film (>30 μm) appears to be uniform in thickness. Figure 8b shows an intermediate image of the film within the area of a hole. Based on the image, the solution grown film shows step changes in its thickness. Figure 8c shows a high magnification image of the film where the atomic structure of MoS_2_ can be observed along the [001] zone axis^31^. The lattice parameter of MoS_2_ measured in the image is 3.2 A, which is in agreement with previous findings^32^.

**Figure 9:**
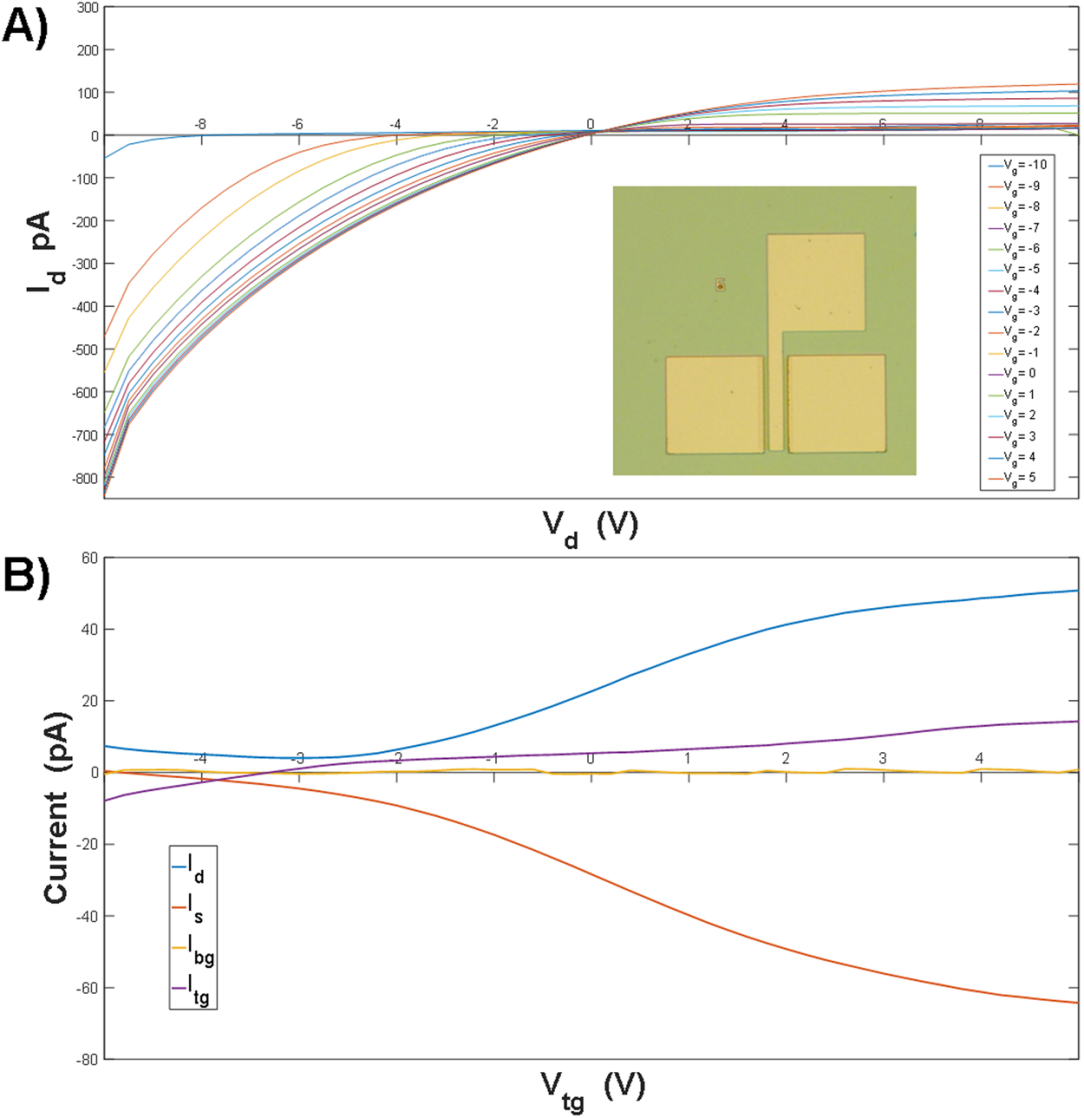
Drain current-drain voltage (**A**) and transconductance (**B**) characteristics for top-gated MoS_2_ field effect transistors.

Double gated MoS_2_ field effect transistors (FET) were fabricated on a highly doped Si (100) wafer with 285 nm thick SiO_2_ dielectric layer using 20 nm and 200 nm Ti and Au contacts. The MoS_2_ film was passivated with a 50 nm thick HfO_2_ layer in order to keep the MoS_2_ film from deteriorating (oxidation) over time from exposure to atmospheric conditions. Measurements were conducted at room temperature in a dark box setup with the bottom gate grounded. Figure 9A shows a series of e I_d_ versus V_d_ curves (I_d_-V_d_) for a top gated MoS_2_ FET with the top gate voltage (V_tg_) ranging from −10V to 5V. Figure 9B shows transconductance curves for the MoS_2_ FET, where three of the plots are the I_d_-V_tg_ curve, the applied I_s_-V_tg_ current profile and the leakage current from the top gate, I_g_-V_tg_. Although there was some leakage current through the top gate, it is apparent that the majority of the current is from the contribution of I_d_-V_tg_. The current through the bottom gate (I_bg_) is also shown to demonstrate that there was no leakage through the bottom gate. The total measured current for both I_d_-V_d_ and I_d_-V_tg_ are on the order of 1 pA, indicative of a resistive MoS_2_ film. The overall resistive behavior could be due to the presence of small domains in the MoS_2_ film, and/or high resistance (non-Ohmic) of the Ti/Au contacts.

## Discussion

Enhanced wetting capability of DMSO-EDTA resulted in a uniform spin-coated layer of the mixture solution on the wafer surface. To assess the effect of protonated carboxylic acid groups on wettability, a solution of tetra-methylated EDTA and ammonium tetrathiomolybdate in DMSO was prepared and spin coated according to the techniques described in the methods section. The resulting sample was found to be too uneven in quality to anneal and barely retained any of the DMSO solution on the wafer surface. Our observations suggest that the protonated carboxylic acid groups play a crucial role in retaining a uniform precursor solution on the silanol functionalized surface.

Further studies on molecular interactions are presented in Fig. 3 where EDTA coordinates MoS_4_^2−^ anions when dissolved in DMSO. Primarily the EDTA/MoS_4_ solution is spin coated onto SiO_2_/Si wafers, spreading the coordinated metal ions evenly over the surface^27^. Heat from the first step of the annealing sequence evaporates DMSO and evolves H_2_S and NH_3_ gases leaving an equivalent of MoS_3_ coordinated by one equivalent of EDTA^19, 33^, which then evaporates at elevated temperatures, leaving MoS_3_ evenly distributed on the silanol functionalized surface. Mo(VI) trisulfide is then reduced to the crystalline Mo(IV) disulfide product and generates S^0^ as a byproduct. The films formed using EDTA solutions were optically uniform, with thicknesses ranging from single-layer to a few-layers. As shown in Fig. 3, the proposed role of EDTA includes: EDTA H-bond formation with DMSO and MoS_4_^2−^ ions; the adsorption of EDTA to the hydroxyl-terminated SiO_2_ substrate; and, coordination with MoS_3_ prior to conversion to MoS_2_. In the as-prepared solution, EDTA forms multiple hydrogen bonds (H-bonds) with both the DMSO solvent molecules and the MoS_4_^2−^ ions. Simultaneously, the surface of the substrate, composed of hydroxyl-terminated SiO_2_, interacts with EDTA via H-bonds. Thus, a layer of EDTA forms on the SiO_2_ surface, with at least one carboxyl group from EDTA interacting with a hydroxyl group from SiO_2_. This can be described as anchoring the precursors to the substrate. Ryczkowski *et al*. identified the interactions between EDTA and γ-Al_2_O_3_ supports via FTIR/PAS measurements^34^. Their studies concluded that there was a high dispersion of Ni metal on the surface of the alumina supports due to interactions of the chelate with the surface hydroxyl groups^34^. The interactions between EDTA and the hydroxyl groups support the idea that EDTA both chelates MoS_4_^2−^ ions and distributes them on the hydroxyl-terminated SiO_2_ substrate surface via an anchoring action. We propose that, with the H-bond network established, ramping to a temperature of 100 C causes DMSO to evaporate, thereby decreasing the concentration of DMSO and increasing the concentration of MoS_4_^2−^, which continues to interact with EDTA. After a film of EDTA-anchored MoS_4_^2−^ ions forms on the substrate, the temperature is then ramped up to 450 C. In this step, the MoS_4_^2−^ converts to MoS_3_ in the temperature range 120–260 C, according to reports by Brito *et al*.^35^. During the annealing step up to ~260 C, EDTA continues to coordinate with MoS_3_ until the EDTA decomposes. The EDTA-MoS_3_ complexation was demonstrated by Badoga *et al*., leading to dispersed MoS_3_ crystallites^27^. Here we demonstrate a similar process with MoS_3_ in the temperature range of 120–260 C, which generated a film of highly dispersed EDTA-anchored MoS_3_ by action of chelation^27^. As the temperature is ramped from 450 C to 800 C, the well-dispersed MoS_3_ converts to MoS_2_. This reaction was previously confirmed by Furimsky and Amberg, who reported the highest degree of crystallinity at 800 C, showing strong x-ray signals for hexagonal MoS_2_^36^. We drew from the aforementioned strategies and chelation-synthesis mechanism to help explain the functionality of EDTA in effectively dispersing the MoS_2_ precursor crystallites (MoS_3_), as well as the reactions that take place at various temperature ranges, which ultimately convert MoS_4_^2−^ to MoS_2_. Thereby, exploitation of the related chelating properties of EDTA has led to a novel contribution to the field of TMD thin film synthesis.

In conclusion, we have demonstrated a novel approach for improving the dispersion of MoS_2_ growth sites by incorporating a common chelating agent, EDTA, with ammonium tetrathiomolybdate in DMSO solvent as a spin-coating solution for MoS_2_ thin film growth. The thickness of the MoS_2_ layers can be tuned depending on the EDTA and thiomolybdate concentrations, and large-area coverage of MoS_2_ is made possible via the chelating action of EDTA with the thiomolybdate ions and subsequent intermediates to MoS_2_. Raman, XPS, and FTIR analysis confirmed the effect of using a chelating agent on MoS_2_ film quality and substrate coverage. Our methods could pave the way towards solution processing of a variety of TMD thin films and their heterostructures at the wafer scale for applications in electronics, optoelectronics and spintronics.

## Methods

### Materials synthesis

Procedures for growing single layer and few-layer MoS_2_ thin films are as follows: First, a 4-inch diameter silicon substrate with a 300 nm thick SiO_2_ film (Si/SiO_2_ substrate) was washed with acetone and IPA for several times, followed by an cleaning in an RCA-1 solution (5:1:1 v/v H_2_O:NH_4_OH:H_2_O_2_) at 100 C for 20 minutes. After a follow up wash in DI H_2_O, the substrate surface was then partially etched using a dilute HF solution (1:5 v/v HF:H_2_O) for 20 seconds. After that, the wafer was cleaned for several times in DI H_2_O, then rinsed in IPA and statically dried. A 5 mL solution of 0.1 M ethylenediaminetetraacetic acid (EDTA) was prepared in Dimethylsulfoxide (DMSO), to which 60 mg of ammonium thiomolybdate ((NH_4_)_2_MoS_4_) (Sigma Aldrich high purity 99.99%) was added and dissolved. The solution was stirred for 2 hours, then filtered with a 0.2 μm polycarbonate filter. Several drops of this solution were dropped onto a cleaned Si/SiO_2_ substrate, followed by spin coating for 1 minute at 3000 rpm to establish a thin and homogenous coating on the substrate. Subsequently, the substrate was placed in a quartz tube furnace for thermal processing, which was purged for multiple times with Argon under 500 Torr vacuum. Then the temperature was ramped from room temperature to 100 C over 30 min and held for 30 min, followed by ramping to 450 C over 30 minutes and held for 60 minutes, and finally ramped to 800 C over 30 minutes and held for 10 minutes. The system was then slowly cooled, and the sample was removed for characterization.

### Transfer

For TEM imaging, samples cleaved from a wafer were first coated with PMMA using a spin coater at 3000 rpm and baked for 5–10 min on hotplate at 100 C. The samples were then placed into a 5% HF solution in order to etch and undercut the SiO_2_ layer to release the grown MoS_2_ films. Samples were then sectioned and fished onto a TEM grid followed by an acetone and chloroform wash, then kept in acetone for at least 30 minutes before drying.

### Materials Characterization

Raman and PL spectroscopy were conducted using a Horiba LabRAM HR instrument with a laser wavelength of 532nm. Raman imaging was performed using a WiTec alpha300R system with a 532 nm light source (333 nm spot size) and a spectral resolution of +/−1 cm^−1^. X-ray photoelectron spectroscopy (XPS) characterization was conducted using a Kratos AXIS ULTRA^DLD^ XPS system equipped with an A1 Kα monochromatic X-ray source and a 165 mm mean radius electron energy hemispherical analyzer along with a vacuum pressure of 3 × 10^−9^ Torr. FTIR measurements were taken using a Nicolet 6700 FTIR system having ATR accessory with a resolution of 0.500 cm^−1^. Scanning transmission electron microscopy (STEM) imaging of the MoS_2_ films were conducted using a FEI Titan G2 60–300 X-FEG aberration-corrected and STEM equipped system with a CEOS DCOR probe corrector. ADF-STEM images (2048 × 2048 pixel^2^) were acquired on the STEM operating at 200 keV using a dwell time of 3–6 µs per image pixel at a camera length of 130 mm. The beam convergence angle *α*_*obj*_ was measured to be 23 mrad. The ADF detector inner and outer angles of collection were measured to be 54 mrad and 317 mrad, respectively. Under these conditions, the measured probe size was ~0.8 Å.
